# Does Identifying with Another Face Alter Body Image Disturbance in Women with an Eating Disorder? An Enfacement Illusion Study

**DOI:** 10.3390/nu17111861

**Published:** 2025-05-29

**Authors:** Jade Portingale, David Butler, Isabel Krug

**Affiliations:** 1 School of Psychological Sciences, The University of Melbourne, Melbourne 3010, Australia; jade.portingale@unimelb.edu.au; 2 Faculty of Psychology and Counselling, The Cairnmillar Institute, Melbourne 3123, Australia; david.butler@cairnmillar.edu.au

**Keywords:** eating disorder, self-perception, face perception, body image disturbance, enfacement illusion, embodiment illusion, multisensory integration

## Abstract

**Background/Objectives**: Individuals with eating disorders (EDs) experience stronger body illusions than control participants, suggesting that abnormalities in multisensory integration may underlie distorted body perception in these conditions. These illusions can also temporarily reduce body image disturbance. Given the centrality of the face to identity and social functioning—and emerging evidence of face image disturbance in EDs—this study examined, for the first time, whether individuals with EDs exhibit heightened susceptibility to a facial illusion (the enfacement illusion) and whether experiencing this illusion improves face and/or body image. **Methods**: White Australian female participants (19 with an ED and 24 controls) completed synchronous and asynchronous facial mimicry tasks to induce the enfacement illusion. Susceptibility was assessed via self-report and an objective self-face recognition task, alongside pre- and post-task measures of perceived facial attractiveness, facial adiposity estimation, and head/body dissatisfaction. **Results**: The illusion was successfully induced across both groups. Contrary to predictions, ED and control participants demonstrated comparable susceptibility, and neither group experienced improvements in face or body image. Notably, participants with EDs experienced increased head dissatisfaction following the illusion. **Conclusions**: These findings indicate that the multisensory integration processes underlying self-face perception, unlike those underlying body perception, may remain intact in EDs. Participant reflections suggested that the limited therapeutic benefit of the enfacement illusion for EDs may reflect the influence of maladaptive social-evaluative processing biases inadvertently triggered during the illusion. A novel dual-process model is proposed in which distorted self-face perception in EDs may arise from biased social-cognitive processing rather than sensory dysfunction alone.

## 1. Introduction

Body image disturbance is a central feature of eating disorders (EDs), particularly anorexia nervosa (AN) and bulimia nervosa (BN) [1], and it is also common in binge eating disorder (BED) and other specified feeding or eating disorders (OSFED) [2]. These disturbances play a central role in the onset and maintenance of EDs and are consistently associated with poorer treatment outcomes [2,3]. While considerable attention has been directed to cognitive-affective disturbances, such as body dissatisfaction and drive for thinness, growing evidence highlights the importance of perceptual distortions in EDs, particularly overestimation of body size and weight [4,5]. These perceptual disturbances are common, persistent, and linked to poorer clinical prognosis [4,6,7], and yet, they remain relatively under-investigated and neglected in conventional treatments [8,9]. The underlying mechanisms driving these distortions, and more broadly, how the bodily self is mentally represented in EDs, remain unclear.

Recent neuroscientific advances consistently demonstrate that bodily self-representations are not static but rather dynamic and continuously shaped through multisensory integration—the process by which the brain combines sensory information across different exteroceptive (e.g., vision and touch), interoceptive, and proprioceptive modalities [10,11]. Experimental paradigms leveraging multisensory integration, namely embodiment illusions, have provided compelling insights into the malleability of bodily self-representation. These illusions temporarily alter one’s sense of bodily self (e.g., body ownership and agency) by inducing the illusory sensation that an artificial body part or entire artificial body is one’s own [12]. For example, in the classic rubber hand illusion, participants observe a rubber hand being stroked while feeling synchronised touches on their unseen hand; such congruent visuo-tactile cues create the illusory sensation of owning the rubber hand [13]. These multisensory principles have been extended to the entire body using full-body illusions which induce ownership over entire artificial bodies, often via virtual reality [14]. Notably, full-body illusions have been successfully induced over virtual bodies that significantly differ from the participant’s own body (e.g., it is larger or slimmer), demonstrating the capacity to experimentally manipulate body image [15,16].

In ED research, embodiment illusions have emerged as powerful tools for probing the multisensory foundations of distorted body experience. By experimentally manipulating the integration of visual, tactile, and proprioceptive signals, these illusions offer compelling evidence that abnormalities in multisensory integration may underlie the altered bodily self-perception characteristic of EDs (for review, see [17]). Studies have consistently shown that individuals with AN and other EDs are more susceptible to the rubber hand illusion compared to healthy controls [18,19,20]. Similar findings have emerged from full-body illusions stimulating the abdomen in AN patients [21]. These results suggest increased malleability or ‘plasticity’ of the bodily self in ED populations, potentially implicating disrupted multisensory integration mechanisms in the development of ED-related body image disturbance. Moreover, embodiment illusions hold therapeutic potential: temporarily adopting another body or body part into one’s body representation---particularly one that differs in perceived physical appearance or aligns with physical appearance ideals (e.g., a ‘healthy-weight’ model)—has been shown to temporarily reduce perceptual body distortions, as well as body dissatisfaction and other related ED symptoms [20,22]. Interestingly, some studies suggest that these improvements are most pronounced in individuals with EDs (compared to controls) or correlate positively with ED symptom severity [21,23], possibly reflecting a greater initial mismatch between internal body representations and external multisensory input—thus allowing more scope for illusion-driven recalibration.

Despite these advances, research on self-perception in EDs has largely focused on the body, neglecting the face. This omission is surprising, given the centrality of the face to self-identity and visual self-recognition, as it typically represents one’s most distinctive physical feature [24,25,26]. The face also plays an important role in social cognition and emotional communication [27,28]. Additionally, facial appearance, especially perceived facial attractiveness and adiposity (i.e., perceived facial weight) is a key target of social evaluation and appearance ideals [29]. The digital age has further intensified facial appearance concerns and dissatisfaction, especially among social media users [30,31]. EDs are associated with disturbances in identity [32,33] and socio-cognitive and socio-emotional functioning [34], and social media usage is a well-documented risk factor for EDs, especially among young women [35]. Yet, empirical investigations into face image disturbance are lacking.

Some studies suggest that women with, or at high risk for, EDs—unlike controls or those at low risk—exhibit impairments in self-face perception, including reduced self-face recognition accuracy [36,37] and disturbances in face image (e.g., greater facial dissatisfaction, lower perceived attractiveness, and elevated adiposity estimation) [37,38]. These findings parallel the well-documented disturbances in body image seen in individuals with EDs, pointing to a broader disruption in self-representation within this population. However, ED research and treatments overwhelmingly focus on body shape and weight (e.g., [8]). Face-specific perceptual distortions are rarely targeted in treatment, and their clinical impact remains poorly understood. Addressing this gap is essential for a more comprehensive understanding of image disturbance and for developing interventions that address the various manifestations of self-related perceptual disturbances in EDs.

One promising experimental paradigm for studying self-face representation is the enfacement illusion [25,26,39], which, like embodiment illusions, manipulates multisensory integration to show that not only the body but also the face self-representation is malleable. In enfacement illusions, synchronous visuo-motor or visuo-tactile stimulation between the participant’s face and that of another individual/model can induce self–other merging, whereby the features of the external face are incorporated into one’s self-face representation [24]. Asynchronous stimulation often serves as a control condition because it seems not to induce the illusion [24]. Importantly, the enfacement illusion thus offers a controlled and powerful tool to study the perceptual plasticity of self-face representation and its underlying multisensory mechanisms. Yet, its application in ED populations is critically limited (for a discussion on this, see [40]).

In a recent community-based study by our team [41] of 226 White and Asian female participants (*n* = 102 high ED-risk based on the Eating Attitudes Test-26 [EAT-26 [42]]), we found no differences in enfacement illusion susceptibility between high and low ED-risk groups. Unexpected post-enfacement illusion effects were also observed as follows: high-risk participants showed increased body and head dissatisfaction, whereas low-risk participants showed reduced body dissatisfaction. These findings broadly contrast with prior embodiment illusion research indicating that participants with a clinical ED or higher levels of ED symptomatology show greater illusion susceptibility and might also derive greater therapeutic benefit from these illusions than controls and those with lower levels of ED symptomatology (e.g., [18,19,20,21,23]). However, community samples may mask clinically meaningful effects. Community-based embodiment illusion studies have reported the null effects of non-clinical ED symptomatology on susceptibility [39] and negative changes in body image post-embodiment that correlated with the degree of (non-clinical) ED symptomatology [43].

While embodiment illusion research has highlighted the role of multisensory integration in body image disturbance, most studies have focused on the body rather than the face. This is a critical omission, given the face’s central role in self-identity, social perception, and contemporary appearance concerns, particularly in populations vulnerable to EDs. Emerging evidence suggests that individuals with EDs or high ED risk show abnormalities in self-face perception, including impaired recognition and heightened dissatisfaction. The enfacement illusion is able to experimentally probe and potentially recalibrate self-face representations via multisensory integration processes. Yet, no prior study has directly examined whether clinical ED populations show altered enfacement illusion susceptibility or perceptual benefits following an enfacement illusion.

To address these gaps, the present study aimed to examine two primary research questions: (1) whether participants with an ED show heightened susceptibility to the enfacement illusion compared to control participants? and (2) whether experiencing the enfacement illusion improves face and/or body image, particularly among those with an ED?

We hypothesised that participants with an ED would be more susceptible to the enfacement illusion than control participants (H1) and that experiencing the illusion would produce improvements in face and/or body image (H2), with greater benefits among participants with an ED compared to controls (H3).

## 2. Materials and Methods

Ethical approval was granted by a Melbourne university (ID: 2056250.1). All participants provided informed consent and were compensated for their time with university course credit or e-gift vouchers. The study followed a published protocol [44] with minor deviations in the clinical sample. The study’s design, hypotheses, and analysis plan were pre-registered on the Open Science Framework.

### 2.1. Participants

A total of 43 female participants (19 with an ED and 24 controls) with a mean age of 24 years (SD: 8.4; range: 18–52) were included in the study. ED and control participants were recruited from a Melbourne university’s participant pool, noticeboards across Melbourne universities, social media, and word-of-mouth referrals. ED participants were also recruited through ED-related organisations and private practices. Inclusion criteria required all participants to be cisgender women, White, ≥18 years old, proficient in English, and without physical conditions preventing task performance.

ED participants were initially identified based on self-reported current ED diagnosis and symptom levels indicative of high ED risk using the EAT-26 [42] and were then required to meet the DSM-5 diagnostic criteria for an ED [45], which was verified using the Eating Disorder Examination (EDE) v17.0D [46]. Participants with comorbid psychiatric conditions were not excluded as this would not have represented a typical ED sample. Control participants were required to self-report no history of an ED and minimal disordered eating symptoms using the EAT-26 [42].

ED diagnoses included AN restricting (*n* = 6), AN binge-purge (*n* = 2), BN (*n* = 2), and OSFED (*n* = 9). OSFED subtypes comprised atypical AN (*n* = 2), low frequency/limited duration BED (*n* = 1), low frequency/limited duration BN (*n* = 4), and purging disorder (*n* = 2). The mean duration of the ED diagnosis was 5.31 years (*SD* = 8.13), with an average age of onset of 22.16 years (*SD* = 9.72). Participants were typically young adults. Average body mass index (BMI) was considered healthy across ED and control participants (six ED participants and four control participants were considered overweight/obese). ED participants were significantly older and more educated than control participants. For complete participant demographic and clinical information, see Table 1.

### 2.2. Stimuli

#### 2.2.1. Stimulation Videos

**Figure 1:**
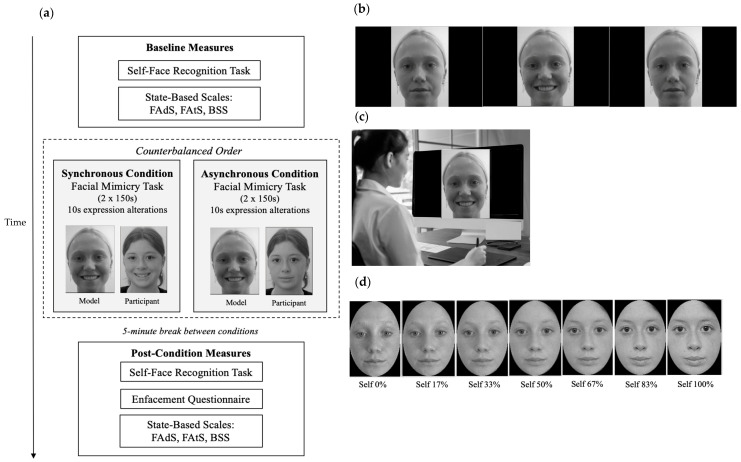
Experimental design, stimuli, and set-up. (**a**) Graphical depiction of the experimental procedure; (**b**) stimulation video depicting a model alternating between neutral and smiling expressions at 10 s intervals, over each 150 s trial (section of the video depicted for illustrative purposes); (**c**) participant completing the synchronous condition (performing the corresponding facial expression to the model); (**d**) morphing video sequence—used for the self-face recognition task—which involved images transitioning from 0% self (100% model) to 100% self (0% model) (depicted in standardised 17% increments for illustrative purposes). Measures: FAds = Facial Adiposity Scale; FAts = Facial Attractiveness Scale; and BSS = Body Satisfaction Scale.

Six young adult White female models were selected based on third-party ratings from a demographically matched sample, ensuring average levels of attractiveness, adiposity, and likability, as well as emotional neutrality—attributes influencing enfacement strength [47] or self-face perception more generally [29]. Videos were recorded of each model to develop the facial mimicry task stimuli used for multisensory (visuo-motor) stimulation (Figure 1b). Each video featured a front-facing model alternating between a neutral expression and an exaggerated visible-tooth smile every 10 s for 150 s—slightly longer than the standard 120 s duration [25] to maximise the likelihood of enfacement.

#### 2.2.2. Morph Videos

For the self-face recognition task (Figure 1d), morphing videos were custom-generated for each participant using a front-view, neutral-expression photograph of their face. Images were mirror-transposed, converted to grey-scale, and circular cropped to remove non-facial features (background, ears, hair) [25] via PhotoScape X (Version 4). Model images underwent the same processing. Each participant’s face was morphed with their model’s face (the same identity used in the stimulation video) in 1% increments, creating a 100-frame video transitioning from 0% to 100% self-face over 100 s using Abrosoft FantaMorph (v5.6.2) [48].

### 2.3. Measures

#### 2.3.1. Demographic and Clinical Information

Self-report demographic information included age, height, weight (to calculate BMI), primary language, sexual orientation, marital status, and employment status. ED history (current/lifetime diagnosis, age of onset, and illness duration) was also collected.

Formal DSM-5 ED diagnoses were verified via the EDE v17.0D [46]. Interviews were conducted by a registered clinical psychologist or trained postgraduate researchers and were double-screened for reliability. The EDE also provides a global severity score based on four subscales (dietary restraint, eating concern, weight concern, and shape concern). The EAT-26 [42], a 26-item measure of ED symptoms, was used to pre-screen the ED group (a validated cut-off score ≥ 20 denoting high ED risk) and determine control group eligibility (a score between 0 and 5 indicating minimal disordered eating). In the present study, internal consistency for the EAT-26 was excellent (α = 0.968).

#### 2.3.2. Subjective Enfacement

The eight-item Enfacement Questionnaire (EQ) [49] assesses subjective enfacement on a seven-point scale (−3 = strongly disagree; 3 = strongly agree). The questionnaire contains three subcomponents as follows: self-identification (four items; feeling that the model’s face was their own), similarity (two items; perceived resemblance), and affect (two items; perceived attractiveness/trustworthiness of the model). Subcomponent scores were calculated by averaging the relevant items (range: −3 to 3), and a total score was computed as the mean of the three subcomponents (range: −3 to 3). Higher scores indicate greater subjective enfacement. In the current study, internal consistency was good across conditions for the total score (α = 0.836–0.844).

#### 2.3.3. Objective Enfacement

In the self-face recognition task [25], participants viewed a morphing video (0% self to 100% self) and were instructed to press the spacebar when the image appeared more like themselves. Stopping times were converted to the % of frames judged as “self”. Earlier stopping times (lower % of self) following stimulation indicate enfacement, reflecting increased attribution of the others’ facial features to the self-face.

#### 2.3.4. Facial Attractiveness and Adiposity

Participants rated their perceived facial attractiveness and adiposity on separate seven-point scales (0 = very unattractive/underweight, 6 = very attractive/overweight). These single-item measures are consistent with widely used methodologies in prior research assessing self-perceptions of facial attractiveness and adiposity (e.g., [50,51]; for a review, see [29]). The psychometric properties of the seven-point Likert scale for facial attractiveness, as used in the present study, have been supported [52]. Research has also shown that single-item Likert-type ratings of facial attractiveness and adiposity are robustly related to objective indices (e.g., BMI for adiposity), supporting their construct validity (for a review, see [29]). Higher scores indicate greater perceived facial attractiveness and adiposity.

#### 2.3.5. Head and Body Dissatisfaction

The Body Satisfaction Scale (BSS) [53] comprises two validated seven-item sub-scales assessing dissatisfaction with features of the head (head, face, jaw, teeth, nose, mouth, and eyes) and body (shoulders, chest, tummy, arms, hands, legs, and feet) (1 = very satisfied; 7 = very unsatisfied). Scores are summed for each subscale (range: 7–49). Higher scores indicate greater dissatisfaction. In the present study, internal consistency was good–excellent across conditions for both subscales (α = 0.894–0.933)**.**

#### 2.3.6. Free-Text Responses

Upon completion of the experiment, participants answered open-ended questions about their experience of the illusion (“What was your overall experience/perception of the enfacement illusion?”) and their appearance perception post-illusion (“How do you feel about your appearance right now?”).

### 2.4. Procedure

Participants were pre-screened for eligibility (ED history and EAT-26) and ED participants completed the EDE v17.0D. Each participant undertook a one-on-one experimental session with a researcher over Zoom, lasting approximately 1.5–2 h, following a structured protocol (see [44] for procedural details). Participants were required to be seated at a laptop or computer positioned directly in front of them, approximately 50 cm away and aligned with eye level.

As depicted in Figure 1a, the session involved a baseline condition comprising a self-face recognition task and state-based body and face image measures. Participants then completed two visuomotor illusion induction conditions in a counterbalanced order. In the synchronous condition, participants observed the stimulation video and mimicked the model’s expressions in real-time (e.g., smiled when the model smiled) (Figure 1b). In the asynchronous condition, participants watched the same video but performed the opposite facial expressions to the model (e.g., smiled when the model was neutral). Each condition involved two facial mimicry trials (150 s per trial), followed by measures of objective and subjective enfacement and then state-based face and body image (randomised). A 5 min break between conditions minimised carryover effects. Model identity was consistent across the stimulation video and self-face recognition task.

After completing both conditions, participants provided free-text responses and demographic information and were debriefed. All questionnaires/scales were delivered via Qualtrics. Self-face recognition and mimicry tasks were administered through PowerPoint, which participants opened locally in full-screen mode and screen-shared for monitoring.

### 2.5. Statistical Analyses

Given the nested data structure (Level 1: repeated measures; Level 2: participants), two multilevel models with random intercepts were tested. Fixed effects included Time (synchronous = 0 asynchronous = 1; synchronous = 0, baseline = 1), Group (control = 0, ED = 1), their interaction (Time × Group), and mean-centred age. Only age was controlled for due to sample size constraints and its established links to ED symptoms [54,55] and enfacement outcomes [26].

The first model tested differential susceptibility to the enfacement illusion (H1), using EQ scores (total and subscales) across synchronous versus asynchronous conditions and self-face recognition task scores across synchronous versus asynchronous and synchronous versus baseline conditions. Stronger enfacement was indicated by higher EQ scores and lower self-face recognition thresholds in the synchronous condition. A Time × Group interaction tested group differences in these changes.

The second model evaluated changes in face and body image post-enfacement—facial attractiveness, facial adiposity (single-item scales), and head/body dissatisfaction (BSS subscales)—comparing synchronous versus baseline conditions (H2). Improvements were defined as increased facial attractiveness and reduced scores on the remaining measures in the synchronous condition. A Time × Group interaction tested group differences in these changes (H3). Only the synchronous versus baseline comparison was examined given prior evidence from embodiment illusion studies that greater body image improvements occur following synchronous compared to asynchronous conditions (for reviews, see [17,56]).

Significant interaction effects were explored via estimated marginal means with Bonferroni-corrected pairwise comparisons. Effect sizes and 95% confidence intervals are reported for all fixed effects. Model fit was assessed using marginal *R*^2^ (fixed effects), conditional *R*^2^ (fixed and random effects) [57], and semi-partial *R*^2^ via the SGV method [58]. Analyses were conducted in R Studio (v4.4.1) using the *lme4* (v1.1.35.5), *lmerTest* (v3.1.3), and *emmeans* (v1.10.5) packages [59,60].

After performing the primary quantitative analyses, participants’ free-text responses were examined descriptively—without formal qualitative coding or thematic analysis—for clear recurring patterns that could help to contextualise or further interpret the quantitative findings. This exploratory analysis was intended solely to enhance understanding of the observed results and was not used to derive or test hypotheses.

#### Power Considerations

An a priori power analysis was not conducted due to the exploratory nature of the study and the limited availability of individuals with clinically verified ED diagnoses. Nonetheless, the final ED sample size (*n* = 19) aligns with the average sample used in comparable embodiment illusion studies in ED populations (for a review, see [17]).

Given the known limitations of post hoc power calculations for multilevel models, including their dependence on observed *p*-values and complex variance structures [61,62], we instead report effect sizes and 95% confidence intervals for all fixed effects [63]. This aligns with current best-practice recommendations in psychological science

## 3. Results

Descriptive statistics for all outcome variables across groups and conditions are presented in Table 2, followed by the results of multilevel models (Table 3) examining susceptibility to enfacement and face and body image changes post-enfacement.

### 3.1. Assumptions Checking

Multicollinearity was within acceptable limits (Variance Inflation Factor scores < 5). Shapiro–Wilk tests indicated normality violations (H2–H3), and residual plots suggested potential heteroscedasticity for one model. To enhance robustness, we applied Satterthwaite approximations for degrees of freedom and computed 95% confidence intervals across models. Several outliers (standardised residuals > 3) were identified, but Cook’s Distance (>4/N) showed no undue influence; all cases were retained to preserve power and sample representativeness. Intraclass correlation coefficients supported the multilevel structure (Table 3).

### 3.2. Susceptibility to Enfacement

#### 3.2.1. Subjective Enfacement

Multilevel modelling analyses revealed a significant main effect of Time for the self-identification subscale of the EQ (semi-partial *R*^2^ = 3%), with higher scores in the synchronous (versus asynchronous) condition, indicating an enfacement effect across the entire sample. Average self-identification scores across conditions fell below the scale’s affirmative range, suggesting such an effect was weak. No significant main effect of Time emerged for the total EQ score or affect and similarity subscale scores (*R*^2^ = 0–1%). No significant main effect of Group (*R*^2^ = 0%) or Time × Group interactions (*R*^2^ = 0–2%) emerged. The models explained 43–67% of the total variance (5–10% unique variance).

#### 3.2.2. Objective Enfacement

A significant main effect of Time (*R*^2^ = 1%) showed lower self-face recognition task scores following synchronous (but not asynchronous) compared to baseline conditions (average reduction of 6.88% of ‘self’ required for self-identification), indicating an enfacement effect across the entire sample. There was no significant main effect for Group or Time × Group interactions (all *R*^2^ = 0%). The model explained 82% of the total variance (4% unique).

### 3.3. Face and Body Image Changes Post-Enfacement

At baseline, ED participants reported greater head and body dissatisfaction, higher perceived facial adiposity, and lower perceived facial attractiveness (near scale extremes) than control participants (around or below scale midpoints).

#### 3.3.1. Facial Attractiveness and Adiposity

No significant main effect of Time or Time × Group interactions emerged for either outcome (*R*^2^ = 0–1%). A significant main effect of Group emerged for attractiveness (*R*^2^ = 12%) but not adiposity (*R*^2^ = 5%), with ED participants reporting lower perceived facial attractiveness than control participants across conditions. The model’s total variance explained was 85% for attractiveness (23% unique) and 75% for adiposity (24% unique).

#### 3.3.2. Head and Body Dissatisfaction

**Figure 2:**
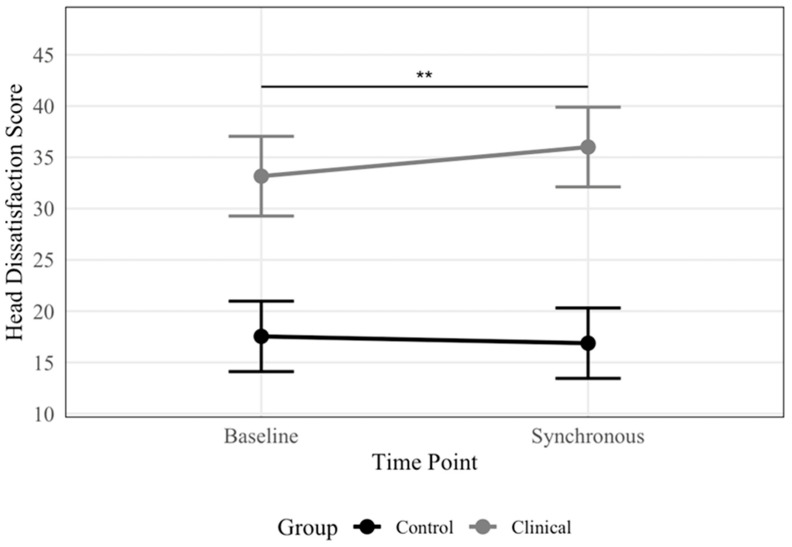
Head dissatisfaction scores by condition and group. The line plot displays the estimated marginal means of head dissatisfaction scores, presented by condition (baseline; synchronous) and group (clinical ED; control). Significantly greater head dissatisfaction was observed following the synchronous condition compared to the baseline condition for the ED group, with no significant difference between conditions for the control group. Error bars represent 95% confidence intervals, with significance brackets showing where differences occur. ** *p* < 0.01.

There was no significant main effect of Time for either outcome (*R*^2^ = 0%) or Time × Group interaction for body dissatisfaction (*R*^2^ = 0%). However, there was a significant Time × Group interaction for head dissatisfaction (*R*^2^ = 1%), indicating that the magnitude of change differed between groups. Post hoc pairwise comparisons (Figure 2) within the ED group revealed significantly greater head dissatisfaction post-synchronous versus baseline conditions (*b* = 2.84, *SE* = 0.91, *t* = 3.12, and *p* = 0.003), with estimated marginal means of 36 and 33 (*SE*’s = 1.9), respectively. No significant timing effects emerged among control participants (*b* = −0.67, *SE* = 0.81, *t* = −0.82, and *p* = 0.415), with estimated marginal means of 17 and 18 (*SE*’s = 1.7) for synchronous and baseline conditions, respectively. The main effect of Group was significant for both outcomes (*R*^2^ = 40–48%), with ED participants reporting greater dissatisfaction than control participants across conditions. The models explained 94% and 98% of the total variance (51–62% unique) for head and body dissatisfaction, respectively.

**Table 2:** Descriptive statistics for outcome variables.

Outcome Variable	Time	Total (*N* = 43)			Eating Disorder (*n* = 19)			Control (*n* = 24)			
		*M*	*SD*	Observed Range	*M*	*SD*	Observed Range	*M*	*SD*	Observed Range	Possible Range
Subjective enfacement											
Total	Sync	−0.22	1.31	−2.67–1.67	−0.38	1.24	−2.67–1.67	−0.09	1.37	−2.67–1.67	−3.00–3.00
	Async	−0.68	1.17	−3.00–1.92	−1.01	1.13	2.67–1.67	−0.42	1.17	−2.67–1.83	
Self-identification	Sync	−0.99	1.53	−3.00–1.75	−1.17	1.56	−3.00–1.75	−0.84	1.52	−3.00–1.75	−3.00–3.00
	Async	−1.53	1.35	−3.00–1.50	−1.57	1.24	−3.00–1.25	−1.50	1.46	−3.00–1.50	
Similarity	Sync	−0.24	1.93	−3.00–2.50	−0.53	1.93	−3.00–2.50	−0.02	1.94	−3.00–2.50	−3.00–3.00
	Async	−0.77	1.80	−3.00–2.50	−1.21	1.72	−3.00–2.50	−0.42	1.82	−3.00–2.00	
Affect	Sync	0.57	1.62	−3.00–3.00	0.55	1.85	−3.00–3.00	0.58	1.45	−2.00–3.00	−3.00–3.00
	Async	0.24	1.42	−3.00–2.50	−0.26	1.61	−3.00–2.50	0.65	1.12	−2.00–2.00	
Objective enfacement	Baseline	60.96	16.28	27.73–95.54	61.42	19.29	41.88–90.00	60.59	13.87	27.73–95.54	0–100
	Sync	54.08	16.91	17.00–96.02	53.66	18.07	27.80–88.00	54.41	16.31	17.00–96.02	
	Async	55.34	16.44	22.54–92.00	56.98	16.46	22.54–92.00	54.04	16.67	29.12–86.49	
Facial attractiveness	Baseline	2.23	1.59	0–5	1.32	1.53	0–5	2.96	1.23	1–5	0–6
	Sync	2.40	1.64	0–5	1.58	1.57	0–5	3.04	1.40	1–5	
	Async	2.26	1.72	0–5	1.53	1.78	0–5	2.83	1.46	0–5	
Facial adiposity	Baseline	3.95	0.97	2–6	4.47	1.07	2–6	3.54	0.66	2–5	1–6
	Sync	3.70	1.15	1–6	4.16	1.38	1–6	3.33	0.76	2–6	
	Async	3.65	1.17	0–6	4.05	1.54	0–6	3.33	0.64	2–5	
Head dissatisfaction	Baseline	24.44	10.94	7–48	32.53	9.13	14–48	18.04	7.53	7–35	7–49
	Sync	25.33	12.03	7–47	35.37	8.19	17–47	17.38	7.91	7–35	
	Async	26.16	12.28	7–48	36.26	9.37	17–48	18.17	7.47	7–32	
Body dissatisfaction	Baseline	28.09	11.54	7–47	38.26	7.02	25–47	20.04	7.17	7–33	7–49
	Sync	28.09	12.59	7–49	39.11	7.88	24–49	19.38	7.83	7–37	
	Async	28.72	12.46	7–49	39.42	7.83	25–49	20.25	8.13	7–41	

Note. *M* = mean; *SD* = standard deviation; sync = synchronous; and async = asynchronous. Objective enfacement scores show the mean % of frames for which the face was perceived as looking more like “self” than “other”.

**Table 3:** Fixed effects results for outcome variables, random effects, and model fit.

Outcome Variable	Fixed Effects	*b*	*b* 95% CI [LB, UB]	*SE*	*df*	*p*	Random Effects (Variance)		Conditional *R*^2 a^	Marginal *R*^2 b^	Semi-Partial *R*^2 c^	ICC
							Intercept	Residual				
Subjective enfacement												
Total	Time: async	−0.33	[−0.84, 0.18]	0.26	41.00	0.212	0.72	0.81	0.51	0.08	0.01	0.47
	Group: ED	−0.18	[−0.96, 0.60]	0.40	63.08	0.655					0.00	
	Age	−0.02	[−0.06, 0.02]	0.02	40.00	0.390					0.01	
	Time: async × ED	−0.30	[−1.07, 0.47]	0.39	41.00	0.445					0.00	
Self-identification	Time: async	−0.66	[−1.15, −0.17]	0.25	41.00	**0.012**	1.39	0.75	0.67	0.05	0.03	0.65
	Group: ED	−0.21	[−1.14, 0.72]	0.48	54.35	0.665					0.00	
	Age	−0.02	[−0.07, 0.03]	0.03	40.00	0.447					0.01	
	Time: async × ED	0.26	[−0.48, 1.00]	0.38	41.00	0.490					0.00	
Similarity	Time: async	−0.40	[−1.12, 0.33]	0.37	41.00	0.292	1.68	1.64	0.55	0.10	0.01	0.51
	Group: ED	−0.19	[−1.34, 0.97]	0.59	61.34	0.754					0.00	
	Age	−0.05	[−0.11, 0.01]	0.03	40.00	0.093					0.05	
	Time: async × ED	0.29	[−1.38, 0.80]	0.56	41.00	0.607					0.00	
Affect	Time: async	0.06	[−0.60, 0.73]	0.34	41.00	0.854	0.90	1.38	0.43	0.07	0.00	0.40
	Group: ED	−0.15	[−1.10, 0.81]	0.49	66.66	0.766					0.00	
	Age	0.02	[−0.03, 0.07]	0.02	40.00	0.443					0.01	
	Time: async × ED	−0.87	[−1.88, 0.12]	0.51	41.00	0.092					0.02	
Objective enfacement	Time: baseline	6.18	[2.02, 10.34]	2.12	82.00	**0.005**	0.91	0.44	0.82	0.04	0.01	0.81
	Time: async	−0.37	[−4.53, 3.78]	2.12	82.00	0.861					0.00	
	Group: ED	−0.35	[−11.14, 10.45]	5.51	50.31	0.950					0.00	
	Age	−0.07	[−0.68, 0.54]	0.31	40.00	0.830					0.00	
	Time: baseline × ED	1.58	[−4.68, 7.83]	3.19	82.00	0.622					0.00	
	Time: async × ED	3.69	[−2.56, 9.94]	3.19	82.00	0.251					0.00	
Facial attractiveness	Time: baseline	−0.08	[−0.45, 0.28]	0.18	41.00	0.654	1.67	0.41	0.85	0.23	0.00	0.80
	Group: ED	−1.51	[−2.43, −0.59]	0.47	47.57	**0.002**					0.12	
	Age	0.01	[−0.05, 0.06]	0.03	40.00	0.781					0.00	
	Time: baseline × ED	−0.18	[−0.72, 0.36]	0.28	41.00	0.521					0.00	
Facial adiposity	Time: baseline	0.21	[−0.10, 0.51]	0.16	41.00	0.190	0.61	0.29	0.75	0.24	0.01	0.67
	Group: ED	0.62	[0.01, 1.22]	0.31	53.26	0.051					0.05	
	Age	0.04	[0.00, 0.07]	0.02	40.00	**0.047**					0.08	
	Time: baseline × ED	0.11	[−0.35, 0.57]	0.24	41.00	0.650					0.00	
Head dissatisfaction	Time: baseline	0.67	[−0.92, 2.25]	0.81	41.00	0.415	57.84	7.88	0.94	0.51	0.00	0.88
	Group: ED	19.13	[13.93, 24.33]	2.66	44.44	**<0.001**					0.40	
	Age	−0.19	[−0.49, 0.11]	0.15	40.00	0.226					0.03	
	Time: baseline × ED	−3.51	[−5.90, −1.12]	1.22	41.00	**0.006**					0.01	
Body dissatisfaction	Time: baseline	0.67	[−0.39, 1.72]	0.54	41.00	0.223	52.38	3.49	0.98	0.62	0.00	0.94
	Group: ED	19.14	[15.85, 25.46]	2.45	42.25	**<0.001**					0.48	
	Age	−0.15	[−0.44, 0.13]	0.14	40.00	0.292					0.03	
	Time: baseline × ED	−1.51	[−3.10, 0.08]	0.81	41.00	0.070					0.00	

Note. CI = confidence interval; LB = lower bound; UB = upper bound; async = asynchronous; ED = eating disorder; *b* = regression coefficient; SE = standard error; and ICC = intraclass correlation coefficient. ^a^ = variance explained by fixed and random effects; ^b^ = shared variance explained by fixed effects; and ^c^ = unique variance explained by each fixed effect. Reference categories (Time = synchronous; Group = control) were assigned a value of 0; whilst categories shown in the table (Time = baseline, asynchronous; Group = ED) were assigned a value of 1. Significant *p*-values (<0.05) are bolded.

### 3.4. Exploratory Insights from Free-Text Responses

An exploratory examination of participants’ open-ended responses suggested that most ED participants (15 of 19; 79%) experienced negative or ambivalent feelings about their appearance following the illusion (e.g., “I feel a bit more negative towards my appearance as I often try to eliminate looking at myself during my day” and “It really highlighted all my flaws”). In contrast, most control participants (21 of 24; 88%) described the illusion as emotionally neutral or mildly positive (e.g., “I felt accepting of my appearance”). Only three control participants (13%) indicated a possible worsening in their perceived appearance post-illusion. Moreover, at least six (32%) ED participants experienced the illusion as a negative, socially evaluative encounter, describing feelings of judgement and self-consciousness (e.g., “I felt really uncomfortable and like the face was judging me” and “When I was mimicking the model I was extremely self-conscious”), while no control participants described a similar negative social experience. Additionally, at least three (16%) ED participants described the morphing task itself as negative or distressing (e.g., perceiving their morphed image as “confronting” or “scary”), while no control participants mentioned distress related to the morphing task.

## 4. Discussion

This study investigated the malleability of self-face representation in EDs and the therapeutic potential of the enfacement illusion, a multisensory integration paradigm previously unexplored in clinical ED samples. The illusion was successfully induced in terms of greater self-reported self-identification with the model and misattribution of the model’s facial features to the self-face (as indexed via earlier self-recognition thresholds in the other-to-self facial morphing task) following synchronous stimulation. Unexpectedly, however, participants with an ED and control participants showed comparable susceptibility to the illusion, and neither group showed improvements in face or body image after experiencing the illusion. Instead, participants with an ED selectively reported *increased* dissatisfaction with their head region.

### 4.1. Enfacement Illusion Susceptibility

Contrary to expectations, ED and control participants showed comparable susceptibility to the enfacement illusion across subjective and objective outcomes, as indexed by self-reported experiences (i.e., self-identification) and performance on the facial morphing task following synchronous stimulation compared to baseline and/or asynchronous stimulation. Such findings of equivalent susceptibility mirror those in our recent community study observing equivalent enfacement susceptibility across low- and high-ED-risk groups [41]. This pattern broadly contrasts with evidence of heightened susceptibility to bodily illusions in clinical ED samples (e.g., [19,21]). The dissociation suggests that facial self-representations, unlike body representations, may retain normative multisensory flexibility in subclinical and clinical ED populations. Such a finding suggests that distortions in self-face perception in ED populations—as observed in reduced self-face recognition accuracy and negative evaluations [31,32]—may arise from mechanisms distinct from those underpinning body distortions. Such a face–body dissociation is broadly supported by neuroimaging evidence suggesting the partial anatomical and functional separation of face and body processing systems [64,65].

The current findings can be contextualised within a predictive coding framework [66,67], according to which self-perception is an inferential process shaped by the interaction between bottom-up sensory inputs and top-down expectations (based on prior knowledge and beliefs within internal mental models). Within this framework, any discrepancy between expected and actual sensory input generates a prediction error, which the brain attempts to resolve either by updating internal models or reinterpreting the sensory data to fit prior beliefs. The strategy employed depends on the relative precision or reliability of each source of information. Embodiment illusions, including the enfacement illusion, are a powerful means to study these dynamics. The enfacement illusion in the present study arises when synchronised visuo-motor stimulation between one’s own and another’s face creates a prediction error that is resolved by incorporating external sensory signals (e.g., the other’s facial features) into one’s self-representation.

Heightened susceptibility to embodiment illusions in individuals with EDs has been interpreted as reflecting an overreliance on rigid and maladaptive prior beliefs about the body at the expense of current sensory input [17,68]—possibly due to known deficits in multisensory integration, namely, an overreliance on visual relative to somatosensory inputs [68]. This imbalance may promote the persistence of distorted body representations in EDs, whereby rigid and maladaptive pre-existing expectations about the body (e.g., beliefs about being overweight) are over-relied upon at the expense of conflicting sensory information [68].

Importantly, while predictive coding frameworks have been applied to explain top-down distortions in bodily representations among individuals with EDs, the current findings suggest that this model may not extend as readily to self-face representations in these conditions. One possible reason is that self-face representations, despite being emotionally salient [37], may be less affected by top-down distortions than bodily representations in EDs. Indeed, body image distortions in EDs tend to be global, affecting the body as a whole, and while some evidence points to localised concerns, these centre more on weight-relevant body regions (e.g., stomach, thighs) than on the face [5,69]. Furthermore, research has shown that individuals with AN direct more visual attention toward body stimuli than to faces during passive viewing tasks (e.g., via eye-tracking) [70], suggesting that the self-face in EDs may be a lesser source of emotional or appearance-based information. Together, these findings suggest that self-face representations might be less affected than the body by top-down distortions in EDs, thus allowing for the normal integration of bottom-up sensory bodily information and resulting in comparable levels of facial malleability (enfacement illusion susceptibility) among ED and control participants.

It is also worth considering the possibility that the enfacement illusion paradigm lacks sensitivity to detect subtle differences in multisensory integration between ED and control participants. In the current study, the subjective experience of enfacement was modest, as indicated by subjective scores generally falling below the scale’s affirmative range—a pattern consistent with previous enfacement research, unlike the typically observed affirmative scores in embodiment research [24]. Since the face, unlike the body, often plays a central role in identity [39] and visual self-face recognition is believed to be a highly practised, socially important skill that continues to improve in early adulthood [71], this may render self-face representations more stable and less amenable to recalibration via brief multisensory stimulation. However, robust direct comparisons have yet to be published on this issue.

### 4.2. Face and Body Image Changes Post-Enfacement

Contrary to expectations, experiencing the enfacement illusion did not improve face or body image in ED or control participants. Instead, participants with an ED reported increased dissatisfaction with their head region after experiencing the enfacement illusion. This finding aligns with the only previous enfacement study in the ED field, which suggested that experiencing the enfacement illusion may exacerbate rather than alleviate head and body dissatisfaction in participants at high risk for an ED [41]. The lack of changes in perceived facial attractiveness, adiposity, and body dissatisfaction should be interpreted cautiously, given potential limitations in measurement sensitivity, as discussed below.

From a neurocognitive perspective, the Allocentric Lock Theory regarding the aetiology of EDs [72,73] offers one compelling explanation for the observed increase in head dissatisfaction. This theory posits that individuals with EDs are ‘locked’ into a rigid and maladaptive (allocentric) representation of the body stored in long-term memory that resists updating from current (egocentric) sensory inputs. In our sample, the subjective experience of enfacement was modest—self-identification ratings fell below the affirmative range—suggesting that self–other boundaries were likely preserved rather than diminished, rendering the task, at least in part, a social interaction rather than a mirror-like experience. In this sense, the illusion was likely insufficient to elicit egocentric updating to ‘unlock’ entrenched maladaptive representations. Instead, it may have inadvertently reinforced these allocentric representations, resulting in increased head dissatisfaction.

Another complementary explanation for the limited therapeutic benefits of the enfacement illusion for ED participants may lie in the interpersonal and socio-emotional dynamics evoked during the illusion. Unlike body-based embodiment illusions, enfacement illusions centre on the face, which typically plays a unique and central role in social cognition and emotional communication (e.g., gaze patterns and facial expressions) [28]. The illusion in the present study required sustained visual engagement with another person’s face, specifically, direct gaze and alternating smiling and neutral facial expressions, which are powerful social cues [28].

For individuals with EDs, such contexts may trigger maladaptive socio-cognitive processes. Although speculative, exploratory insights from participants’ open-ended reflections revealed that most ED participants reported heightened appearance concerns after the illusion, and many reported affective and interpersonal discomfort with the model’s gaze during the illusion, including feelings of judgment and self-consciousness. In contrast, control participants predominantly described the illusion as emotionally neutral or even positive.

While informal, these observations align with theoretical models of self-focused attention [74], particularly within the context of EDs. This construct comprises two key dimensions as follows: private self-consciousness (awareness of one’s internal thoughts and feelings) and public self-consciousness (concern with how one is perceived by others). Prior experimental research suggests that exposure to another person’s face can enhance social self-focused attention in participants with high levels of ED symptoms, heightening their focus on exteroceptive information about their body (i.e., physical appearance) rather than interoceptive body information [75]. In this sense, the enfacement illusion task, which required participants to maintain constant visual engagement with a stranger’s face (600 s in total), may have inadvertently heightened social self-consciousness in ED participants, thereby intensifying focus on perceived appearance flaws.

Moreover, this socio-evaluative framing may help to explain why head dissatisfaction increased specifically. Faces are not only important for self-identity but also uniquely sensitive to social mirroring as follows: one’s self-face perception is not just visual (e.g., mirror-based), but shaped by social feedback and beliefs about how others perceive oneself (e.g., by observing others facial expressions in response one’s own face) [76]. For individuals with EDs, such beliefs may be negatively biased, especially under conditions of heightened social self-consciousness. Eye-tracking studies have shown that individuals with EDs, especially AN, avoid visually attending to faces, especially those that are emotionally expressive [70,77], which is believed to reflect discomfort with social interactions including a fear of social evaluation [77]. Indeed, individuals with EDs frequently show negative social evaluative biases, including heightened sensitivity to appearance-based rejection, which often manifests as misinterpreting ambiguous or neutral social cues as signals of rejection [34,78]. In this sense, even simple social (affective) cues like watching a model’s face alternating between smiling and neutral expression might have been misinterpreted as signals of appearance-based rejection. Ultimately, this may have reinforced negative perceptions about one’s head or face as the body region on display, ultimately, increasing head dissatisfaction.

In sum, although speculative, our findings raise important questions about the limits of multisensory interventions for EDs when social-evaluative processes remain unaddressed. In this sense, self-face perception in individuals with EDs may even be driven by a deeper unease with self–other dynamics, with negative self-face evaluations emerging primarily in contexts that trigger social self-consciousness.

It is also worth considering that task design factors, namely, administering the facial morphing task before measuring face/body image within each condition, may have inadvertently influenced shifts in participants’ appearance-based self-evaluations independently of the illusion. Since individuals with EDs show identity disturbances [32,33], blurring self–other boundaries and reducing self-perceptual coherence, at least to some extent, during the facial morphing task (albeit at a level no different from controls) might have invertedly heightened discomfort in ED participants, ultimately increasing their head/face dissatisfaction. Such interpretation is speculative; however, it is supported by some post-experiment ED participant reflections that described their morphed image negatively (e.g., as ‘mutant’ and ‘confronting’), whilst no control participants reported the same negative response.

Interestingly, the heightened dissatisfaction was specific to the head. One possibility is that while the body holds more entrenched cognitive-affective salience in EDs, the face becomes more salient in social contexts that provoke interpersonal evaluation. The null effects on facial attractiveness and adiposity may reflect poor measurement sensitivity, as these outcomes were assessed with single-item scales. In contrast, the head dissatisfaction scale, as a composite measure of multiple facial features, may have better detected localised appearance concerns. Individuals with EDs, especially AN, display a feature-focused visual bias away from salient facial features (e.g., eyes, mouth) [77]. Since these regions convey social information (e.g., emotion, gaze), avoidance may reflect anxiety around social judgement. Over time, this pattern may reinforce associations between these regions and perceived scrutiny, inadvertently increasing dissatisfaction with specific aspects of the face.

### 4.3. Strengths and Limitations

Study strengths include the clinically verified ED sample, reducing misclassification biases common in embodiment illusion research (for review, see [17]) and a comprehensive battery of face and body evaluation measures alongside controlled comparisons between synchronous and asynchronous stimulation conditions, enhancing internal validity. While the sample size was modest, it is consistent with prior embodiment studies in clinical ED populations [79,80,81]. Nonetheless, without an a priori power analysis, the sample may have been underpowered to detect subtle effects or interactions, particularly given the small effect sizes observed (1–3% unique variance explained). Given the sample size and model complexity, we also acknowledge the potential for Type II error, particularly for interaction effects. Future studies with larger samples are recommended to more robustly detect subtle group differences.

Additionally, including a mixed ED sample reflects clinical reality, given symptom overlap across diagnoses [82], and aligns with previous embodiment research [18,19,23]. Nonetheless, this approach may have obscured diagnosis-specific effects, especially as multisensory integration abnormalities are better established in AN [68]. Future research should employ larger, diagnostically stratified samples and ensure sufficient power to account for relevant covariates such as BMI and alexithymia.

Further limitations might include the single-item measures of facial attractiveness and adiposity, which may not adequately capture the complexity of self-face perception. While these measures are supported by prior research and show strong correlations with objective indices (e.g., BMI) [29], future studies should consider including more comprehensive and multidimensional assessments. This may include assessing specific facial attributes such as symmetry, proportionality, and region-specific evaluations (e.g., [83]), although such instruments require psychometric validation and hence have not yet been widely adopted. In addition, incorporating objective measures—such as eye-tracking indices of visual attention or facial anthropometric markers like the facial width-to-height ratio (see [29])—could provide more precise insights into the nature and manifestation of self-face perceptual disturbances in individuals with EDs.

Additionally, the use of ‘average’ faces may have confounded responses; attractive faces are known to strengthen enfacement [47], yet, if self–other boundaries are preserved during enfacement, incorporating attractive faces may provoke adverse outcomes in ED populations (e.g., via upward appearance comparisons [84]). Tailoring facial stimuli to individual social-affective profiles, such as by enhancing model likeability or reducing perceived social threat, could improve therapeutic utility.

Furthermore, whilst the present study utilised insights from informal descriptive analyses of free-text responses, future researchers should conduct formal qualitative analyses of participants’ experiences during the illusion and appearance evaluations post-experiment to gain deeper insights.

Finally, findings may not generalise beyond White cisgender women. Given that cultural and gender differences in body and face image are well-documented [85,86], future enfacement research should examine whether enfacement effects differ across diverse populations (for a discussion, see [87]). For instance, cultural schemas may shape the domains (face versus body) around which ED-related perceptual biases consolidate.

### 4.4. Proposed Dual-Process Model of Self-Face Perception in Eating Disorders

**Figure 3:**
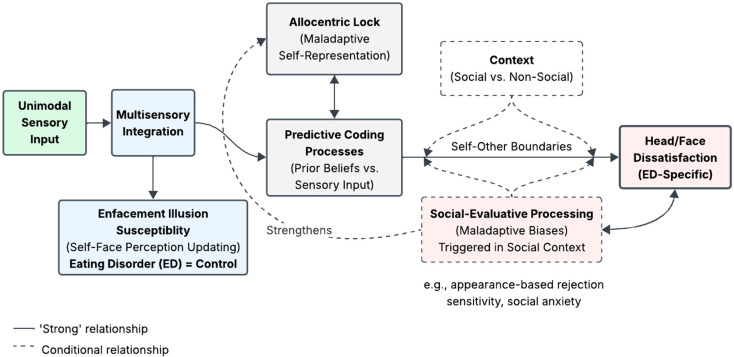
Self-face perception in eating disorders: proposed dual-process model.

Pending replication and extension, the current findings, considered within the context of broader literature and participant reflections, highlight the potentially complex and context-dependent nature of self-face perception in individuals with EDs and may offer implications for theory and practice. Accordingly, Figure 3 provides a schematic representation of the proposed dual-process model of self-face perception in EDs. Within this model, self-face perception emerges through a hierarchical process beginning with unimodal sensory processing, followed by multisensory integration, and culminating in predictive coding processes that reconcile incoming sensory input with existing prior beliefs. This predictive framework also interacts with spatial frames of reference (allocentric versus egocentric processing) and is modulated by contextual factors, particularly the social versus non-social nature of the perceptual experience.

In individuals with EDs, our findings suggest a dual-process model whereby multisensory integration underlying enfacement susceptibility remains unaffected, resulting in comparable illusion susceptibility across ED and control groups. However, for individuals with an ED, downstream self-face perception may become compromised through context-dependent pathways. Specifically, when self-face perception occurs within social contexts—where self–other boundaries are preserved due to weak illusory effects and exposure to social-emotional cues (e.g., eye gaze and facial expressions)—maladaptive top-down beliefs and social-evaluative processing biases in individuals with EDs may trigger social self-consciousness (e.g., judgement fears and interpersonal discomfort), resulting in head/face dissatisfaction. In this sense, the affective consequences of the illusion may have arisen from the interplay between incoming sensory input (e.g., seeing the model’s face) and rigid, maladaptive prior beliefs regarding social evaluation (e.g., “others judge my facial appearance negatively”). Moreover, following the allocentric lock hypothesis, the lack of egocentric updating of self-representations may reinforce maladaptive allocentric self-representations. Ultimately, these processes may create a reinforcing cycle whereby head/face dissatisfaction strengthens the allocentric lock and social-evaluative biases, perpetuating self-face perceptual disturbances.

### 4.5. Future Research Directions and Clinical Implications

Future research should prioritise directly testing the proposed dual-process model by disentangling the perceptual, social-affective, and cognitive dimensions of self-face perception in EDs. Specifically, ED-relevant traits, such as alexithymia, autism spectrum characteristics [88,89], social anxiety, and rejection sensitivity [34,78], should be systematically examined. These factors may influence how individuals with EDs experience self–other overlap—e.g., alexithymia and autism spectrum characteristics have been shown to influence embodiment illusion susceptibility (either attenuating or strengthening it) outside of ED contexts [90]—or whether these individuals interpret the illusion as an emotionally charged social encounter that drives social self-consciousness.

Future researchers should also investigate how individuals with EDs perceive social evaluation, particularly beliefs about being judged due to their ED and discomfort with their face being seen or judged, and importantly, assess whether/how these priors influence enfacement outcomes. Additionally, future researchers should examine whether self-face representations in EDs are indeed less susceptible to multisensory recalibration due to their weaker emotional salience, for instance, or whether other factors are at play; for example, such representations only become distorted within certain social contexts.

Future studies should also critically examine the morphing task itself, which—despite its widespread use [24]—may not constitute a purely objective measure of self-recognition, at least in individuals with an ED. Qualitative reflections from ED participants tentatively suggest that the task may inadvertently elicit affective discomfort or identity disruption, potentially influencing self-evaluations in real-time. In future studies, integrating qualitative methods could enhance sensitivity to individual differences in task experience,

Clinically, the current observation that head dissatisfaction increased after experiencing enfacement for participants with EDs prompts caution against enfacement-based interventions for EDs that do not account for the social dynamics embedded in the task. Nonetheless, these findings do not discourage future research into enfacement-based interventions for EDs. Prior research supports the potential for the enfacement illusion to recalibrate and improve face and/or body image in individuals at low ED risk [41], suggesting that it may have preventive or subclinical applications. Such effectiveness in the early stages of ED development may be due to maladaptive allocentric representations having not yet become entrenched (see Allocentric Lock Theory [72,73]) or because social-evaluative processing biases are not attenuating the illusion’s therapeutic potential. Instead, for clinical ED groups, enfacement-based interventions may need to be adapted, such as by combining them with emotionally supportive cues (e.g., autobiographical memory recall to unlock allocentric representations [91]) or by adapting these approaches to individual differences (e.g., socio-emotional processing). The latter approaches might include incorporating features that promote social safety and emotion regulation (e.g., non-judgmental facial expressions, averted gaze, or cognitive reappraisal techniques) or reducing the social significance of the illusion (e.g., using avatars instead of real models).

## 5. Conclusions

Although future research is needed to replicate and extend our findings, evidence of equivalent enfacement illusion susceptibility between individuals with EDs and control participants underscores a potential dissociation between how multisensory processes contribute to face versus body image disturbance in EDs. However, for individuals with EDs, self-face perception may specifically become compromised through context-dependent pathways, specifically in social contexts due to social-evaluative processing biases and social self-consciousness. In this sense, while enfacement illusions may not offer universal therapeutic benefits for individuals with EDs, it may not be the illusion’s perceptual strength that matters most, but rather how the experience is interpreted on a socio-cognitive or socio-emotional level.

This study proposes a novel dual-process model for understanding self-face perception in individuals with EDs. Future researchers should directly investigate how interpersonal processing shapes self-perception in EDs and how enfacement interventions may be better tailored to enhance their therapeutic potential.

## Figures and Tables

**Table 1:** Participant demographic and clinical characteristics.

Variable	Group				
	ED (*n* = 19)	Control (*n* = 24)	Total (*N* = 43)	*t*/*χ*^2^	*p*
Age (*M* ± *SD*)	27.47 ± 10.70	21.50 ± 4.77	24.14 ± 8.40	−2.45	**0.019**
BMI (*M* ± *SD*)	21.15 ± 7.00	22.60 ± 3.55	21.96 ± 5.34	0.89	0.381
Primary language spoken at home (*n*, %)				2.55	0.110
English	19 (100.0%)	21 (87.5%)	40 (93.0%)		
Other	0 (0.0%)	3 (12.5%)	3 (7.0%)		
Highest education completed (*n*, %)				11.18	**0.048**
Year 12 or below	7 (36.8%)	15 (62.5%)	22 (51.2%)		
Certificate/diploma	4 (21.0%)	0 (0.0%)	4 (9.4%)		
Bachelor’s degree	5 (26.3%)	4 (16.7%)	9 (20.9%)		
Postgraduate degree (e.g., Honours, Masters, PhD)	3 (15.8%)	5 (20.8%)	8 (18.6%)		
Sexual orientation (*n*, %)				4.13	0.388
Heterosexual	11 (57.9%)	16 (66.7%)	27 (62.8%)		
Lesbian/gay	1 (5.3%)	0 (0.0%)	1 (2.3%)		
Bisexual	4 (21.1%)	6 (25.0%)	10 (23.3%)		
Asexual	2 (10.5%)	0 (0.0%)	2 (4.7%)		
Other	1 (5.3%)	2 (8.3%)	3 (7.0%)		
Marital status (*n*, %)				3.51	0.320
Single	11 (57.9%)	12 (50.0%)	23 (53.5%)		
Relationship (including open relationship)	4 (21.1%)	8 (33.3%)	12 (27.9%)		
Married	2 (10.5%)	0 (0.0%)	2 (4.7%)		
De facto	2 (10.5%)	4 (16.7%)	6 (14.0%)		
ED diagnosis type (yes, %) ^a^				-	-
Anorexia nervosa (restricting)	6 (31.6%)	-	-		
Anorexia nervosa (binge-purge)	2 (10.5%)	-	-		
Bulimia nervosa	2 (10.5%)	-	-		
OSFED ^b^	9 (47.4%)	-	-		
ED diagnosis duration (years) (*M* ± *SD*)	5.31 ± 8.13	-	-	-	-
Average age of ED onset (years) (*M* ± *SD*)	22.16 ± 9.72	-	-	-	-
EDE interview score (*M* ± *SD*)				-	-
Global severity	4.02 ± 0.99	-	-		
Restraint	3.99 ± 1.26	-	-		
Eating concern	3.12 ± 1.13	-	-		
Weight concern	4.87 ± 0.94	-	-		
Shape concern	4.56 ± 1.12	-	-		
EAT-26 score (*M* ± *SD*)	46.58 ± 13.05	2.79 ± 1.56	22.14 ± 23.63	−14.54	**<0.001**

Note. BMI = Body Mass Index (kg/m^2^); ED = eating disorder; OSFED = other specified feeding or eating disorder; *M* = mean; *SD* = standard deviation; EDE = Eating Disorder Examination interview v 17.0D [46]; and EAT-26 = Eating Attitudes Test-26 item questionnaire [42]. *t*-test for continuous variables, chi-squared test for categorical variables. ^a^ ED diagnosis type verified via the EDE interview. ^b^ OSFED types included atypical anorexia nervosa (*n* = 2), binge-eating disorder (of low frequency or limited duration) (*n* = 1), bulimia nervosa (of low frequency or limited duration) (*n* = 4), and purging disorder (*n* = 2). Significant (two-sided) *p*-values are bolded.

## Data Availability

The datasets analysed during the current study are not publicly available due to ethical restrictions related to participant confidentiality, but are available from the corresponding author upon request.
